# External beam irradiation of myocardial carcinoid metastases: a case report

**DOI:** 10.1186/1752-1947-1-95

**Published:** 2007-09-19

**Authors:** Jonathan Strosberg, Sarah Hoffe, Todd Hazelton, Larry Kvols

**Affiliations:** 1 H. Lee Moffitt Cancer Center and Research Institute, 12902 Magnolia Drive, Tampa, FL 33612, USA

## Abstract

The heart is an exceedingly rare site of metastatic involvement in carcinoid tumors. Only nineteen cases have been described in the literature over the past 30 years. We report here on a patient who presented with progressive carcinoid syndrome despite surgical resection of her liver metastases. She was found to have cardiac metastases on inidium-111-pentetreotide scintigraphy and subsequently underwent external beam radiation to the heart resulting in symptomatic palliation of her syndrome and objective radiographic response. To our knowledge, this is the first reported case of metastatic cardiac carcinoid treated with external beam irradiation.

## Case presentation

A 45 year-old woman with a history of metastatic carcinoid disease presented to our institution with progressive flushing and diarrhea.

She had been diagnosed with a carcinoid tumor 18 months earlier when an enlarged mesenteric lymph node was discovered incidentally during laparoscopic gastric bypass surgery. Pathologic evaluation demonstrated a moderately differentiated neuroendocrine tumor. Laboratory studies revealed an elevation in 24 hour urine 5 hydroxyindoleacetic acid (5HIAA) of 37.4 mg (reference range 1–6 mg) and an elevated serum serotonin of 1671 ng/ml (reference range 22–180 ng/ml). At the time of initial diagnosis, the patient denied any prior history of flushing, diarrhea or wheezing.

**Figure 1:**
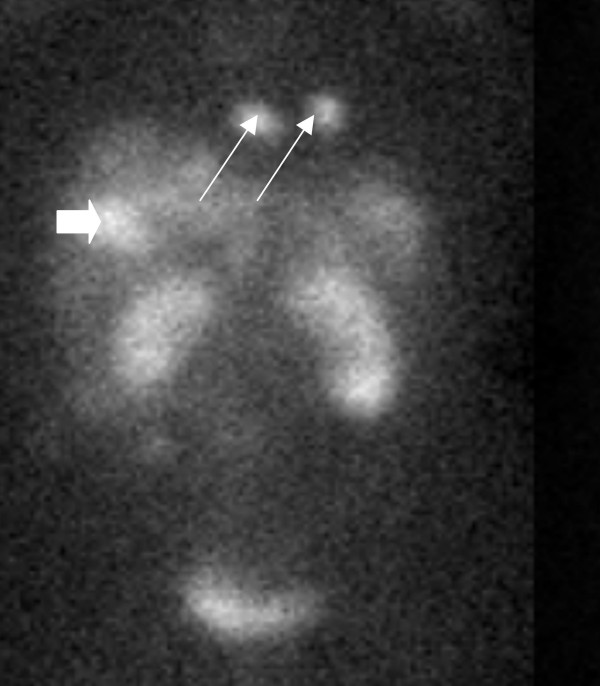
In-111-Pentetriotide Scan (OctreoScan) demonstrating a liver metastasis (block arrow) as well as two distinct tumors in the region of the heart (thin arrows).

Magnetic resonance imaging of the abdomen revealed two hepatic masses, one in the dome of the liver measuring 6.8 cm, and another in the posterior right hepatic lobe measuring 2.2 cm. Indium-111-pentetreotide scintigraphy (OctreoScan™) revealed a focus of activity in the right hepatic lobe, as well as two foci of intense activity in the cardiac region (figure 1). A primary gastrointestinal or bronchial tumor was not detected.

Depot octreotide therapy was initiated, and a series of three laparoscopic hepatic radiofrequency ablations were performed over the ensuing 6 months. Although this therapy resulted in normalization of the 24-hour urine 5-HIAA, serum serotonin remained elevated (539 ng/ml). Moreover, the patient began developing progressive diarrhea and intense facial flushing despite dose escalation of depot octreotide from 30 mg to 60 mg every 4 weeks.

Repeat indium-111-pentetreotide scintigraphy twelve months after initial diagnosis demonstrated resolution of activity in the liver. However, persistent radiotracer uptake was detected in the region of the myocardium. Echocardiography was ordered to evaluate for the presence of cardiac metastases. It demonstrated trace to mild tricuspid insufficiency and trace aortic insufficiency, but showed no evidence of an intracardiac mass.

**Figure 2:**
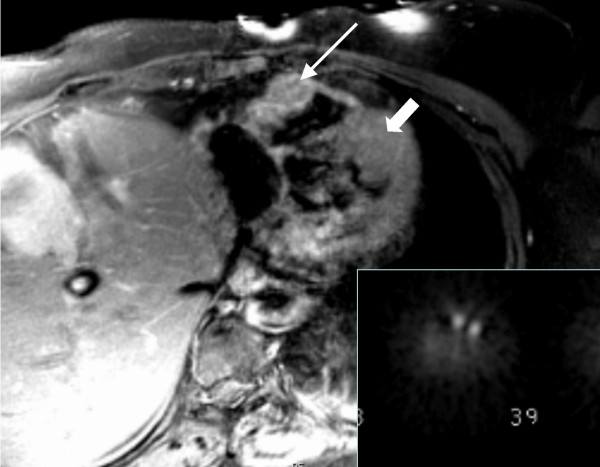
T1 gated axial MRI image demonstrating mass in the right ventricular wall (long arrow) and more poorly-defined lesion in the left ventricular wall (block arrow) corresponding to sites of abnormal radiotracer uptake on indium-111-pentetreotide scintigraphy (inset).

Magnetic resonance (MR) imaging of the chest was performed using multiplanar breath-hold, electrocardiogram-gated HASTE (Half Fourier Single Shot Turbo Spin-Echo) and electrocardiogram-gated, post-gadolinium T1-weighted spin-echo images obtained on a 1.5-Tesla Symphony MR scanner (Siemens Medical Systems, Malvern, PA). The MR images demonstrated a 3.8 × 2.1 cm mildly enhancing mass involving the free wall of the right ventricle as well as a poorly-defined mass involving the mid anterior left ventricular wall with extenstion to the apex. (Figure 2). These masses correlated to sites of abnormal radiotracer accumulation on indium-111-pentetreotide scintigraphy (Figure 2 inset).

**Figure 3:**
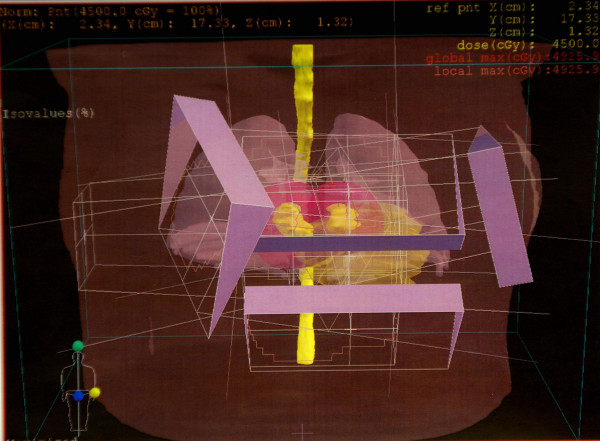
3-dimensional representation of fields with wedges.

**Figure 4:**
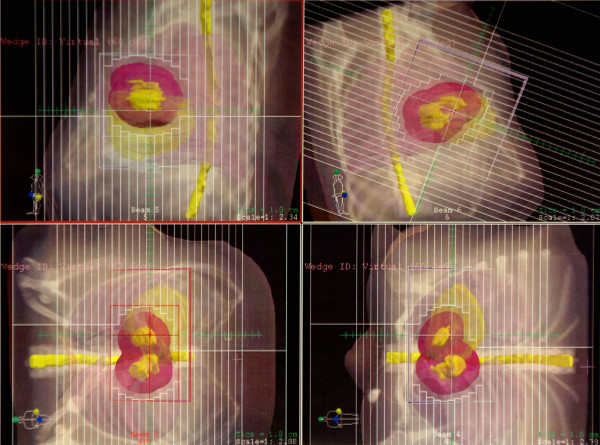
Multileaf collimator treatment fields.

Consideration was given to surgical resection of the myocardial metastases, however, the risk of malignant arrhythmias was deemed prohibitive. Therefore, radiation oncology was consulted for external beam irradiation. A total of 45 Gray was delivered to the myocardium in 25 fractions using 3D-conformal technique (figure 3). The patient's gross tumor volume (GTV) in both the right and left ventricles was drawn on the planning CT scans that had been taken with the patient in the supine position with her arms immobilized over her head in a vac-lok bag. The MRI images were used for aid in target volume delineation. Four beams consisting of an anterior superior oblique (ASO), anterior inferior oblique (AIO), left anterior superior oblique (LASO), and a right anterior superior oblique (RASO) were planned to deliver 45 Gy to the GTV + a 2 cm margin (figure 4). Dose volume histogram (DVH) analysis showed that with this beam arrangement, <40% of the heart received the prescribed dose of 45 Gy, 60% of the heart received 30 Gy, and 50% received 40 Gy. 30% of the total lung volume received 20 Gy. 100% of the GTV received the prescription dose. Treatment was delivered using 18 MV photons with 60 degree wedges on each field. The therapy was complicated only by a brief episode of chest pain and dyspnea without electrocardiographic changes.

**Figure 5:**
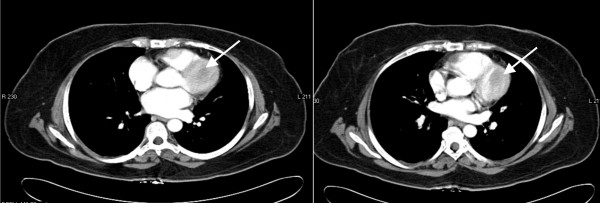
Left ventricular mass before (5.a) and after (5.b) external beam irradiation.

Repeat magnetic resonance imaging after completion of radiation demonstrated a fifty percent bidimensional reduction in the size of the right ventricular tumor (figure 5) associated with a substantial decrease in serum serotonin levels to 205 ng/ml. Six months after treatment, the patient denied any chest pain, dyspnea or orthopnea and reported a moderate improvement in her facial flushing and diarrhea. Interferon α was added to octreotide therapy for further management of her metastatic disease.

## Discussion

Although right sided heart valve fibrosis and dysfunction is a hallmark of carcinoid heart disease, myocardial metastases from carcinoid tumors are distinctly uncommon. Among 243 patients undergoing echocardiography for evaluation for carcinoid heart disease, sonographic evidence of cardiac tumor was observed in only 6 patients (2%).[1] Another series of 132 patients undergoing echocardiograms for evaluation of carcinoid heart disease demonstrated a 4% rate of myocardial metastases.[2]

While echocardiography has been the primary technique for identifying myocardial metastases from carcinoid tumors, other methods have also been described in the literature. Puvaneswary et al. described the use of magnetic resonance imaging for localization of cardiac metastases[3], while Yeung et al. reviewed the use of ^111^Indium-pentetreotide scintigraphy.[4] In this case, scintigraphy demonstrated radiotracer uptake in the region of the heart while MRI scans provided important anatomic information enabling use of focused radiation therapy. Echocardiography, however, failed to accurately identify the cardiac tumors.

The location of cardiac metastases in prior reported cases has included all chambers of the heart as well as pericardium.[1,2,5,6] Two patients presented with malignant arrhythmias, including one patient who developed fatal bradycardia due to tumor infiltration of the atrioventricular node.[7] Carcinoid syndrome associated with elevation in urine 5HIAA has been documented in the majority of cases described in the literature.

An interesting feature of this case was the persistence of elevated serotonin along with progressive flushing and diarrhea despite the successful resection of liver metastases through radiofrequency ablation and normalization of urine 5 hydroxyindolacetic acid (5HIAA). Since inactivation of serotonin and its catabolism into 5HIAA occurs partially in the lung, we hypothesize that the direct secretion of serotonin into the systemic circulation by the left ventricular metastasis may have resulted in exacerbation of the carcinoid syndrome despite ablation of liver metastases.

Surgical resection of cardiac metastases has been the therapy of choice in the majority of carcinoid tumors described in the literature.[1,2,4] A minority of cases were treated with systemic chemotherapy.[5] The use of external beam irradiation for control of cardiac metastases from carcinoid tumors has not been described in the literature but there have been single institutional series reporting the effectiveness of radiation for other sites of metastatic disease.

Schupak and Wallner reviewed 44 patients with metastatic or unresectable carcinoid tumors who were treated at Memorial Sloan-Kettering Cancer Center with radiation.[8] The sites included in their series were epidural space, brain, bone, and abdomen. Infield control following radiotherapy was 77% for epidural sites and 78% for bone sites. No patient with brain metastasis had progression of intracranial disease after radiation and 80% of patients with abdominal disease achieved a complete or partial response. The authors did not demonstrate a dose response relationship and recommended 45–50 Gy in 4–5 weeks for non-hepatic sites. Chakravarthy and Abrams reported on 18 patients with metastatic or unresectable carcinoid patients at Johns Hopkins.[9] In their series, clinical improvement occurred in 87% of the sites irradiated.

Cardiac effects of radiation have been evaluated in great detail, with data derived largely from follow-up of long term survivors of Hodgkin's disease. Current evidence has shown that radiation to the heart can be associated with pericardial disease, accelerated atherogenesis of the coronary arteries, myocardial disease, valve defects, and predisposition to conduction abnormalities.[10-13]

## Conclusion

The heart is a rare site of involvement in metastatic carcinoid tumors. Electrocardiogram-gated MRI may be a useful imaging modality for anatomic delineation of the tumor. Carcinoid tumors involving the myocardium can produce substantial morbidity due to invasion of sensitive cardiac structures and also due to secretion of serotonin directly into the systemic circulation. When surgical resection is considered excessively risky, external beam irradiation may be an effective option for palliation of symptoms. The potential benefits need to be weighed against the risks of pericardial, valvular and myocardial toxicity as well as chronic atherogenesis.

## Competing interests

The author(s) declare that they have no competing interests.

## Authors' contributions

SH commented on radiation technique and safety. TH commented on radiologic modalities and interpretation. JS and LK prepared the remainder of the manuscript including clinical history and therapeutic outcome.
